# Association Between Cardiovascular Disease Risk Factors and Mortality in Adults With Diabetes: A Stratified Analysis by Sex, Race, and Ethnicity

**DOI:** 10.3389/ijph.2022.1604472

**Published:** 2022-04-06

**Authors:** Kristina Savage, Joni S. Williams, Emma Garacci, Leonard E. Egede

**Affiliations:** ^1^ Center for Advancing Population Science (CAPS), Medical College of Wisconsin, Milwaukee, WI, United States; ^2^ Division of General Internal Medicine, Department of Medicine, Froedtert & The Medical College of Wisconsin, Milwaukee, WI, United States

**Keywords:** mortality, race, ethnicity, sex, diabetes, cardiovascular disease risk factors, adults

## Abstract

**Objectives:** To assess sex and racial/ethnic differences in the relationship between multiple cardiovascular disease (CVD) risk factors and mortality among a nationally representative sample of adults with diabetes.

**Methods:** Data were analyzed from 3,503 adults with diabetes from the National Health and Nutrition Examination Survey 2001–2010 and its linked mortality data through 31 December 2011. The outcome was mortality; the independent variables were sex and race/ethnicity. Covariates included demographics, comorbidity, and lifestyle variables. Cox proportional hazards regression was used to test associations between mortality and CVD risk factors.

**Results:** In adjusted analyses, the association between diastolic blood pressure and mortality was significantly different by sex and race/ethnicity (unadjusted *p* = 0.009; adjusted *p* = 0.042). Kaplan-Meier survival curves showed Hispanic women had the highest survival compared to Hispanic men and Non-Hispanic Black (NHB) and Non-Hispanic White (NHW) men and women; NHW men had the lowest survival probability.

**Conclusion:** In this nationally representative sample, stratified analyses showed women had higher survival rates compared to men within each race/ethnicity group, and Hispanic women had the highest survival compared to all other groups.

## Introduction

More than 34 million Americans have diabetes, which represents 10.5% of the US population (1). Of all diabetes cases, 90–95% are classified as being type 2 diabetes mellitus (T2DM) (1). The national prevalence of diabetes has been on the rise for decades, and the number of adults with the disease is projected to increase to 60.6 million, or 17.9% by 2060, with the highest prevalence existing among minorities (2, 3). The prevalence of diabetes by sex differs by race and ethnicity with American Indian/Alaska Native (AI/AN) and Non-Hispanic Black (NHB) women having a higher prevalence of diabetes compared to AI/AN and NHB men, respectively (1). Hispanic, Non-Hispanic Asian, and Non-Hispanic White (NHW) men have a higher prevalence of diabetes compared to women of the same race and ethnicity.

Sex and racial/ethnic differences among individuals with T2DM may contribute to disparity trends observed in mortality (4–7). Evidence suggests women with T2DM have less favorable CVD risk factor profiles compared to men, and fewer women achieve target levels for hemoglobin A1c (HbA1c), low-density lipoprotein cholesterol (LDL-C), and systolic blood pressure (SBP) (4, 8–10). While men with T2DM have a higher absolute risk of mortality (2, 11), the increased risk factor burden observed in women with diabetes is thought to contribute to a significantly increased relative risk of CVD and mortality (9, 13, 14). In women with diabetes, the cardioprotective effect of the female sex is attenuated (15, 16).

Similarly, compared to NHWs, racial/ethnic minorities have poorer control of CVD risk factors (6, 7, 17). While there has been an overall increase in CVD risk factor control between 2001–2009, NHBs display less control of HbA1c, BP, LDL-C, and composite control of all three factors compared to NHWs (7). Furthermore, national data indicate that NHBs and Hispanics are 2.3 and 1.5 times more likely to die, respectively, from poorly controlled T2DM compared to NHWs (18). However, while national mortality rates among racial/ethnic minorities with diabetes are higher compared to NHWs, many studies have shown a “reverse disparity” in the relationship between race/ethnicity and mortality where NHWs display a higher mortality risk (19, 20) Therefore, the aim of this study is to assess sex and racial/ethnic differences in the relationship between multiple CVD risk factors and mortality among a nationally representative sample of adults with diabetes.

## Methods

This was a cohort study that analyzed 2001–2010 data from the National Health and Nutrition Examination Survey (NHANES) and its linked mortality data through 31 December 2011. NHANES is a survey program that combines interviews and physical examinations from a nationally representative sample of adults and children to assess the health and nutritional status of the United States. We investigated disparities in mortality by race/ethnicity, sex, and the influence of key modifiable risk factors among persons with diabetes.

### Study Population

The study population included men and women aged 20 years and older, who completed both the survey interview and Mobile Exam Center (MEC) examination and had mortality follow-up information. In total, 26,269 participants were selected. Among them, 3,664 participants were identified as having diabetes; we excluded 161 other minorities and 3,503 were included in the analyses.

### Diabetes Classification

Participants with diabetes were defined based on their responses to diabetes-related questions and their measured HbA1c. Participants were considered to have diabetes if they answered “yes” to any of the following three questions: “Have you ever been told by a doctor or other health professional that you have diabetes or sugar diabetes?”; “Are you now taking insulin?”; or “Are you now taking diabetic pills to lower your blood sugar?”; or if they answered “no” to all three questions, but had a measured HbA1c≥6.5%.

### Mortality Outcome

The survey data were linked to the most recently available mortality data, the public-use 2011 Linked Mortality File. All survey participants were followed from interview date through to 31 December 2011. Mortality outcome of interest in these analyses included all-cause mortality.

### Cardiovascular Disease Risk Factors

Traditional CVD risk factors including LDL-C, HbA1c, SBP, diastolic blood pressure (DBP), and body mass index (BMI) were assessed in this study. BMI was classified as normal weight (<25 kg/m^2^), overweight (25–29 kg/m^2^), obese (30–39 kg/m^2^), and morbidly obese (40+ kg/m^2^). SBP was defined as normal blood pressure (<120 mmHg), prehypertension (120–139 mmHg), stage 1 hypertension (140–159 mmHg), and stage 2 hypertension (≥160 mmHg). DBP was defined and categorized as normal blood pressure (<80 mmHg), prehypertension (80–89 mmHg), stage 1 hypertension (90–99 mmHg), and stage 2 hypertension (≥100 mmHg). LDL-C was defined and classified as normal (<130 mg/dl), borderline (130–159 mg/dl), and high (≥160 mg/dl). HbA1c was categorized into <6.5%; 6.5–6.9%; 7.0–7.9%; and ≥8.0%.

### Covariates

Covariates included demographics, comorbidity, and lifestyle variables. Demographic variables included age and ratio of family income to poverty. Comorbidity was defined as count of the following diseases: congestive heart failure, coronary heart disease, angina, heart attack, stroke, and cancer. It ranged from 0 to 6. Lifestyle variables included physical activity and smoking status. Physical activity, based on the individual’s engagement in work and recreational activities, was categorized as vigorous/moderate and none. Smoking was grouped as never/former or current smoker.

### Statistical Analysis

National Center for Health Statistics (NCHS) recommendations were followed in the analyses to account for the complex survey design. Statistical Analysis System (SAS) procedures for survey sampling were used to calculate correct standard errors (SEs) and p-values. To correct unequal probability sampling bias and nonresponse bias, MEC exam weights were used to represent the US population for BMI, SBP, DBP, and HbA1c outcomes; fasting weights were used for the LDL-C outcome. Survey Cox proportional hazards regression models were used to calculate mortality. All statistical analyses were performed with SAS version 9.4 (SAS Institute).

Cox proportional hazards regression analysis was used to test the association between CVD risk factor and mortality stratified by sex and race and ethnicity for individuals with diabetes. Five CVD risk factors were explored separately. First, each CVD risk factor was treated as continuous; univariate and multivariable Cox regression was modelled for each sex and race interaction. Multivariable models were adjusted for demographics (age, ratio of family income to poverty as continuous), comorbidity (continuous), and lifestyle variables [physical activity (none vs. vigorous/ moderate) and smoking status (current smoker vs. former smoker/none smoker)]. Second, these models were also tested by treating CVD risk as categorical variable.

## Results

The weighted sample population characteristics for adults with diabetes are presented in Table 1 by sex and race/ethnicity. A total of 3,503 participants (representing 19,434,189 US non-institutionalized residents) over the age of 20 years had diagnosed or undiagnosed diabetes and had mortality data available through 2010. Approximately 50.2% were females. While the majority of the population was NHW (66.4%), 18.1% were NHB, and 15.5% were Hispanic. Approximately 45.4% of the sample was between the age of 45–64 years, where the average age was 59 years old. Approximately 55.4% had at least a high school education. Approximately 42.2% were not married, and 23.3% earned below the poverty level based on 130% or less ratio of family income to the poverty line. Approximately 35.7% of the sample had 1 or more comorbidities, where 50.5% reported no physical activity and 53.5% were current or former smokers.

**TABLE 1:** Weighted Sample Characteristics for Adults with Diabetes (sample *n* = 3,503). (National Health and Nutrition Examination Survey, United States, 2001-2010).

Variables	All	NH White	NH Black	Hispanic	P Value
Cohort Weighted Count	19,434,189	12,904,638	3,524,110	3,005,441	
Sex					<0.001*
Men	49.8%	52.3%	40.4%	50.3%	
Women	50.2%	47.7%	59.6%	49.7%	
Age group					<0.001*
20–44 years	15.8%	12.5%	18.5%	26.4%	
45–64 years	45.4%	42.6%	51.5%	50.2%	
65+ years	38.8%	44.8%	29.9%	23.4%	
Education level					<0.001*
High school or below	55.4%	49.7%	62.8%	71.2%	
College or above	44.6%	50.3%	37.2%	28.8%	
Marital status					<0.001*
Married	57.8%	61.8%	43.6%	57.3%	
Not Married	42.2%	38.2%	56.4%	42.7%	
Ratio of family income to poverty					<0.001*
130% and less of poverty level	23.3%	18.1%	29.5%	37.8%	
Above 130% of poverty level	69.2%	75.3%	61.6%	51.7%	
Unknown	7.6%	6.6%	8.8%	10.5%	
Comorbidity group					<0.001*
Comorbidity count = 0	64.3%	59.2%	67.5%	82.7%	
Comorbidity count = 1	20.2%	22.5%	19.3%	11.2%	
Comorbidity count≥2	15.5%	18.3%	13.2%	6.1%	
Physical activity					<0.001*
None	50.5%	48.2%	54.9%	55.0%	
Moderate	32.0%	34.6%	28.3%	25.3%	
Vigorous	17.5%	17.2%	16.8%	19.7%	
Smoking status					<0.001*
None Smoker	46.5%	44.3%	49.2%	52.9%	
Current Smoker	18.7%	17.1%	23.2%	20.6%	
Former Smoker	34.8%	38.7%	27.6%	26.5%	

* Significance at *p* < 0.05.

Table 2 shows the mean values of CVD risk factors for adults with diabetes by sex and race/ethnicity. NHB women had highest mean BMI (35.5 ± 0.5), and Hispanic men had lowest (30.6 ± 0.4) (*p* < 0.001). NHB women had highest mean SBP (134.5 ± 1.1%), and NHW men had lowest mean SBP (127.8 ± 0.9) (*p* < 0.001). NHB and Hispanic men had the highest mean DBP (73.3 ± 1.1 and 73.4 ± 0.8, respectively), and NHW women had the lowest (64.8 ± 0.7) (*p* < 0.001). NHB men had highest mean LDL-C (119.0 ± 3.7), and NHW women had the lowest (109.9 ± 2.6) (*p* < 0.001). Hispanic men had the highest mean HbA1c at 8.0%, and NHW women had the lowest at 6.9% (*p* < 0.001).

**TABLE 2:** Mean values of cardiovascular disease risk factors for adults with diabetes stratified by race/ethnicity and sex. (National Health and Nutrition Examination Survey, United States, 2001-2010).

Variable	NH White	NH Black	Hispanic	NH White	NH Black	Hispanic	P Value
	Men	Men	Men	Women	Women	Women	
Cohort Count	6,743,857	1,425,465	1,512,895	6,160,781	2,098,645	1,492,545	
BMI (kg/m^2^)	32.2 (0.3)	33.0 (0.4)	30.6 (0.4)	33.7 (0.4)	35.5 (0.5)	32.9 (0.5)	<0.001*
SBP (mmHg)	127.8 (0.9)	132.2 (0.9)	128.0 (1.0)	131.4 (0.9)	134.5 (1.1)	128.9 (1.3)	<0.001*
DBP (mmHg)	69.3 (0.7)	73.3 (1.1)	73.4 (0.8)	64.8 (0.7)	69.9 (0.8)	68.5 (0.8)	<0.001*
LDL-C (mg/dl)	101.1 (2.1)	119.0 (3.7)	110.6 (3.1)	109.9 (2.6)	111.0 (2.2)	112.3 (4.0)	<0.001*
HbA1C (%)	7.2 (0.1)	7.8 (0.1)	8.0 (0.2)	6.9 (0.1)	7.5 (0.1)	7.7 (0.2)	<0.001*

* Significance at *p* < 0.05. All numbers represent mean (standard error). Abbreviations: BMI, body mass index; SBP, systolic blood pressure; DBP, diastolic blood pressure; HbA1c, hemoglobin A1c; LDL-C, low-density lipoprotein-cholesterol; kg/m^2^, kilograms/meter squared; mmHG, millimeters of mercury; mg/dL, milligram/deciLiter.

Table 3 shows the unadjusted results of the survey Cox proportional hazard models for the relationship between CVD risk factors and mortality stratified by race/ethnicity and sex in adults with diabetes. BMI was significantly associated with lower mortality in Hispanic men (HR = 0.87, 95% CI 0.78–0.98). SBP was significantly associated with a higher risk of mortality in Hispanic men (HR = 1.02, 95% CI 1.01–1.04), NHW women (HR = 1.01, 95% CI 1.01–1.02), NHB women (HR = 1.02, 95% CI 1.01–1.03), and Hispanic women (HR = 1.02, 95% CI 1.01–1.03). DBP was significantly associated with lower mortality in NHW men (HR = 0.97, 95% CI 0.96–0.98) and NHW women (HR = 0.98, 95% CI 0.97–0.99). HbA1c was significantly associated with lower mortality in NHB women (HR = 0.82, 95% CI 0.68–0.99). LDL-c was not associated with mortality by race/ethnicity or sex in adults with diabetes in the unadjusted models.

**TABLE 3:** Unadjusted cox proportion hazard models for cardiovascular disease risk factors and mortality stratified by race/ethnicity and sex for adults with diabetes. (National Health and Nutrition Examination Survey, United States, 2001-2010).

Predictor	All	NH White	NH Black	Hispanic	NH White	NH Black	Hispanic	Interaction
		Men	Men	Men	Women	Women	Women	P Value
Continuous								
Body Mass Index (kg/m^2^)	0.98 (0.96–1.00)	1.00 (0.96–1.04)	0.99 (0.95–1.03)	**0.87 (0.78–0.98)**	0.98 (0.95–1.01)	0.98 (0.94–1.04)	0.93 (0.86–1.01)	0.381
Systolic Blood Pressure (mmHg)	**1.01 (1.01–1.02)**	1.01 (1.00–1.02)	1.00 (0.98–1.02)	**1.02 (1.01–1.04)**	**1.01 (1.01–1.02)**	**1.02 (1.01–1.03)**	**1.02 (1.01–1.03)**	0.155
Diastolic Blood Pressure (mmHg)	**0.98 (0.97–0.98)**	**0.97 (0.96–0.98)**	0.99 (0.98–1.01)	0.99 (0.96–1.02)	**0.98 (0.97–0.99)**	0.99 (0.98–1.00)	**0.98 (0.96–1.00)**	**0.009** *
Low Density Lipoprotein-Cholesterol (mg/dl)	1.00 (0.99–1.00)	1.00 (0.99–1.01)	0.99 (0.98–1.01)	1.00 (0.99–1.01)	1.00 (0.98–1.01)	0.99 (0.98–1.01)	1.01 (0.99–1.03)	0.791
Blood Hemoglobin (HbA1C) (%)	0.96 (0.91–1.03)	1.00 (0.89–1.13)	1.00 (0.86–1.16)	1.08 (0.86–1.35)	0.98 (0.88–1.09)	**0.82 (0.68–0.99)**	1.08 (0.92–1.26)	0.250

* Bold values indicate significance at *p* < 0.05. Abbreviations: HbA1c, hemoglobin A1c; kg/m^2^, Kilograms/meter squared; mmHG, millimeters of mercury; mg/dL, milligram/deciLiter; NH, non-hispanic.

Table 4 shows the adjusted results of the survey Cox proportional hazard models for the relationship between CVD risk factors and mortality stratified by race/ethnicity and sex in adults with diabetes. BMI was significantly associated with lower mortality in Hispanic men (HR = 0.87, 95% CI 0.79–0.96). SBP was significantly associated with a higher risk of mortality in Hispanic men (HR = 1.02, 95% CI 1.01–1.03). DBP was significantly associated with lower mortality in NHW men (HR = 0.97, 95% CI 0.96–0.98). HbA1c and LDL-C were not associated with mortality by race/ethnicity or sex in adults with diabetes after adjusting for relevant covariates.

**TABLE 4:** Adjusted cox proportion hazard models for cardiovascular disease risk factors and mortality stratified by race/ethnicity and sex for adults with diabetes. (National Health and Nutrition Examination Survey, United States, 2001-2010).

Predictor	All	NH White	NH Black	Hispanic	NH White	NH Black	Hispanic	Interaction
		Men	Men	Men	Women	Women	Women	P Value
Continuous								
Body Mass Index (kg/m^2^)	0.99 (0.97–1.01)	1.00 (0.96–1.04)	0.99 (0.95–1.04)	**0.87 (0.79–0.96)**	0.99 (0.96–1.03)	0.99 (0.94–1.04)	0.95 (0.87–1.03)	0.338
Systolic Blood Pressure (mmHg)	**1.01 (1.00–1.01)**	1.00 (0.99–1.01)	1.00 (0.99–1.02)	**1.02 (1.01–1.03)**	**1.01 (1.00–1.02)**	1.01 (1.00–1.02)	**1.02 (1.00–1.03)**	0.427
Diastolic Blood Pressure (mmHg)	**0.98 (0.98–0.99)**	**0.97 (0.96–0.98)**	1.00 (0.98–1.02)	1.00 (0.97–1.04)	0.99 (0.97–1.00)	1.00 (0.99–1.01)	0.99 (0.97–1.01)	**0.042**
LDL-Cholesterol (mg/dl)	1.00 (0.99–1.01)	1.00 (0.99–1.01)	1.00 (0.99–1.01)	1.00 (0.99–1.01)	1.00 (0.99–1.02)	0.99 (0.98–1.01)	**1.01 (1.00–1.03)**	0.720
Blood Hemoglobin (HbA1c) (%)	1.05 (0.97–1.12)	1.08 (0.97–1.20)	1.03 (0.91–1.17)	1.11 (0.90–1.38)	1.05 (0.90–1.23)	0.81 (0.64–1.02)	1.13 (0.96–1.33)	0.100

*Bold values indicate significance at *p* < 0.05. Abbreviations: HbA1c, hemoglobin A1c; kg/m^2^, kilograms/meter squared; mmHG, millimeters of mercury; mg/dL, milligram/deciLiter; NH, non-hispanic.

**FIGURE 1:**
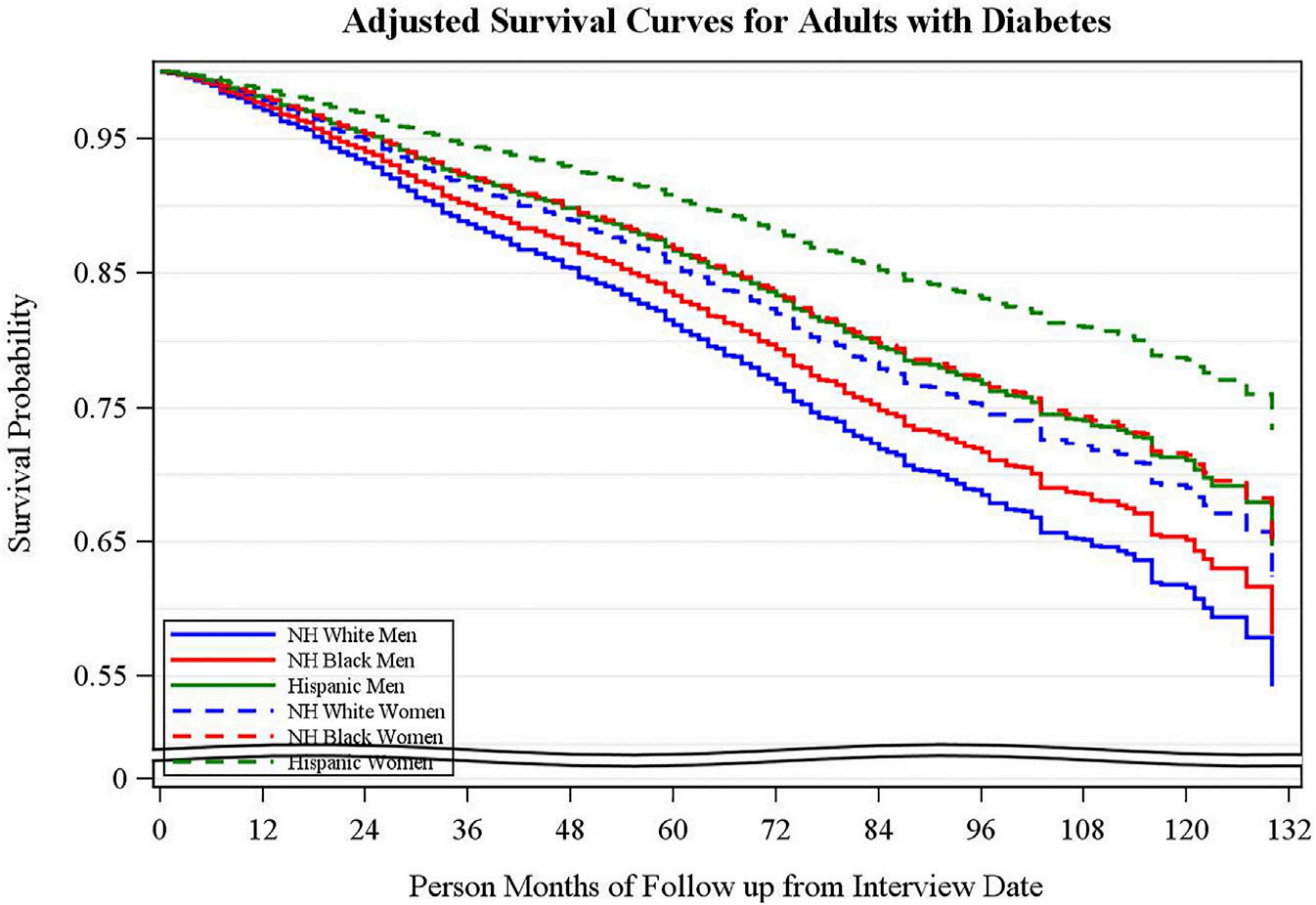
Survival curves for adults with diabetes stratified by race/ethnicity and sex. Adjusted survival curves using the corrected group prognosis method adjusted by age, PIR, comorbidity, smoking status, physical activity. (National Health and Nutrition Examination Survey, United States, 2001-2010).

Adjusted survival curves of adult diabetes population are shown in Figure 1. Hispanic women with diabetes had the highest survival, and NHW men with diabetes had the lowest survival (NHW men compared with Hispanic women *p* = 0.001).

## Discussion

In this sample of adults with diabetes, a significant relationship was found between CVD risk factors and mortality stratified by sex and race/ethnicity, adjusting for relevant confounding factors. In unadjusted Cox Proportion Hazard regression analyses, DBP was significantly associated with a lower risk of mortality in both NHW men and women and Hispanic women. However, after adjusting for relevant covariates, the relationship between mortality and CVD risk factors for NHW and Hispanic women no longer persisted; the relationship remained significant only for NHW men. In addition, adjusted survival curves demonstrated Hispanic women to have the highest survival probability compared to all other racial/ethnic groups; NHW men had the lowest survival probability. There were no statistically significant race/ethnicity and sex interactions for relationship between HbA1c, SBP, LDL-C or body mass index (BMI) and mortality. These findings suggest DBP control should be a goal for management in adults with diabetes, especially among NHW men, to decrease the risk of mortality. Additionally, research is needed to understand the factors associated with higher survival probability in Hispanic women.

Our findings are supported by evidence from previous studies evaluating the relationship between CVD risk factors and mortality. Our study found a significant relationship between DBP and mortality, such that DBP was associated with lower mortality risk. Our findings are supported by Vamos et al., who indicated that in some cases, patients with T2DM and uncontrolled DBP had lower risk of mortality (21). Specifically, they found that in patients with T2DM and CVD, an uncontrolled DBP of 85–89 mmHg was associated with a lower mortality risk (21). Previous studies have reported an increased mortality risk with elevated SBP and DBP in patients with diabetes (22, 23). In their study of NHB and NHW patients with T2DM, Li et al. found that DBP ≥100 mmHg was associated with an increased risk of mortality among both NHB and NHW men and women with diabetes (23). However, the authors also reported that the lowest risk of mortality was associated with a DBP of 80–90 mmHg (23). Similarly, Kontopantelis et al. reported that in patients with T2DM, the lowest risk of mortality was associated with a DBP of 82.5–87.5 mmHg (24). The association between DBP and lower mortality risk found in our study for NHW men suggests more NHW men have a DBP that is in the hypertensive, yet potentially protective range.

Additionally, the results of our study indicated that Hispanic women had the highest survival compared to NHB and NHW racial groups, which could be explained by the “Hispanic paradox,” or the observation that in the US, the mortality rates for Hispanics remain below NHWs despite lower socioeconomic status (SES), worse disease profiles, and increased risk and prevalence for some diseases (25, 26). Explanations for this phenomenon include low smoking prevalence, protective sociocultural practices (e.g., dietary practices, social support, family cohesion, etc.), and the selective migration of immigrants with significant health issues back to their home countries (26, 27). Another reason for this advantage is a mortality rate that is two-thirds that of NHWs for the leading causes of death—heart disease, cancer, and lung disease—among Hispanics (25). Finally, we also observed that women had higher survival rates compared to men in every race/ethnicity category. This finding is supported by other studies that found higher mortality rates and mortality risk in men with diabetes compared to women with diabetes (11, 12, 28). For example, using nationally representative data, Gregg et al. investigated trends in death rates among adults with and without diabetes between 1997 and 2006. Overall, they found that men with diabetes had higher mortality rates compared to women with diabetes (28).

Evidence suggests controlling modifiable cardiovascular disease (CVD) risk factors that include diabetes, hypertension, dyslipidemia, and obesity reduce morbidity and mortality in patients with T2DM (29–31). Maintaining target levels for CVD risk factors among patients with diabetes is an important standard of care given the significant negative health outcomes associated with uncontrolled risk factors such as the development of microvascular and macrovascular complications and mortality (32). In addition, there is evidence that diabetes confers an independent risk for the development of atherosclerotic CVD, which includes coronary heart disease, cerebrovascular disease, and peripheral arterial disease of atherosclerotic origin (33–35). In the US, improved risk factor control has been accompanied by significant improvements in mortality among individuals with diabetes (36). However, despite these improvements, individuals with diabetes still have increased risk of CVD and mortality compared to individuals without the disease (11, 37). Findings from this study provide additional information for targeted interventions to address sex and racial/ethnic disparities and reduce mortality among adults with T2DM.

There are study limitations that should be mentioned. First, in using cross-sectional data, we were unable to determine causality. Second, there are potential confounders not accounted for in the analyses including acculturation, length of diabetes duration, diabetes knowledge, and self-management practices. These factors may have influenced the findings; however, we are not able to substantiate their impact. Third, the sample size for some of the minority racial/ethnic groups was limited and smaller in comparison to the majority group. Finally, given the structure of the national dataset, we were unable to distinguish between T2DM and other types of diabetes including Type 1 Diabetes. Although 95% of adults with diabetes have T2DM, we recognize that individuals younger than 30 years, especially those being treated with insulin, may have Type 1 Diabetes. We were not able to distinguish those individuals from adults with T2DM, so this may have impacted the results.

### Conclusion

The results of our study are important because they provide new information about the relationship between CVD risk factors and mortality by sex and race/ethnicity. In this nationally representative sample, significant associations were found in this relationship: DBP was associated with lower mortality risk in NHW men, and Hispanic women had the highest survival probability, after adjusting for relevant confounders. These results suggest the need for increased investigation and targeting of specific CVD risk factors, clinically, that influence mortality risk in men and women with diabetes across racial and ethnic groups. More studies are needed to determine the mechanisms driving the association between DBP and mortality in NHW men. Finally, additional research is needed to understand the underlying mechanisms associated with increased survival probability in Hispanic women with diabetes.

## Data Availability

The dataset generated and analyzed during the current study is publicly available at https://meps.ahrq.gov/data_stats/download_data_files.jsp.
